# Real-world evaluation of costs of illness for pneumonia in adult patients in Dubai—A claims database study

**DOI:** 10.1371/journal.pone.0256856

**Published:** 2021-09-01

**Authors:** Sara Ahmad Mohammad Al Dallal, Mohamed Farghaly, Ahmed Ghorab, Mostafa Elaassar, Hammam Haridy, Nancy Awad, Badarinath Chickballapur Ramachandrachar, Ashok Natarajan

**Affiliations:** 1 Health Economics & Insurance Policies Department, Dubai Health Authority, Dubai, UAE; 2 Health & Value and Patient Outcomes, Pfizer, Dubai, UAE; 3 Vaccines, Medical & Scientific Affairs, Pfizer, Dubai, UAE; 4 Real World Evidence, IQVIA, Dubai, UAE; University of South Carolina College of Pharmacy, UNITED STATES

## Abstract

**Background:**

Pneumonia is a significant cause of morbidity and mortality among adults globally. This retrospective cohort analysis assessed the pneumonia burden and related healthcare resource utilization and costs in the at-risk (low, medium, and high-risk) adult patients in Dubai, United Arab Emirates (UAE).

**Methods:**

The claims data from January 1, 2014 to June 30, 2019 were extracted from the Dubai Real-World Claims Database for patients, aged ≥18 year, having at least 1 pneumonia claim. Data for the inpatient, outpatient and emergency visits were assessed for 12-months, before (pre-index) and after (follow-up) a pneumonia episode. Healthcare costs were calculated based on dollar value of 2020.

**Results:**

Total 48,562 records of eligible patients were analyzed (mean age = 39.9 years; low [62.1%], medium [36.2%] and high [1.7%] risk cohorts). Mean all-cause healthcare costs were approximately >45% higher in the follow-up period (1,947 USD/patient) versus pre-index period (1,327 USD/patient). During follow-up period, the mean annual pneumonia incidence rate was 1.3 episodes, with a similar pattern across all cohorts. Overall, mean claims and costs (USD) per patient (all-cause) were highest in the high-risk cohort in the follow-up period (claims: overall, 11.6; high-risk, 22.0; medium-risk, 13.9; low-risk, 9.9; costs: high-risk, 14,184; medium-risk, 2,240; low-risk, 1,388). Similarly, the mean pneumonia-related costs (USD) per patient were highest for the high-risk cohort (overall: 1,305; high-risk, 10,207; medium-risk, 1,283; low-risk, 882), however, the claims were similar across cohorts (claims/patient: overall: 2.0; high-risk, 1.9; medium-risk, 2.2; low-risk, 1.9). Most all-cause and pneumonia-related costs were due to inpatient visits (4,901 and 4,818 USD respectively), while outpatient (1,232 and 166 USD respectively) and emergency visits (347 and 206 USD respectively) contributed significantly lesser.

**Conclusions:**

Pneumonia imposes a significant healthcare burden in the UAE, especially in the high-risk patients with severe comorbidities. These findings would guide clinicians and policy makers to make informed decisions.

## Introduction

Pneumonia is an infectious lung disorder that affects people of all ages [1, 2]. It is characterized by inflammation of the lung parenchyma and presents a wide spectrum of clinical symptoms, ranging from mild fever, cough, to severe respiratory distress and sepsis [1–3]. Pneumonia can be caused by various pathogens, among which *Streptococcus pneumoniae* and *Haemophilus influenzae* are the two most common causative microorganisms, that account for the majority of pneumonia-related deaths [4, 5]. Overall, an estimated 450 million pneumonia episodes are reported each year globally [6], with majority of the disease burden contributed by the developing countries [7, 8]. As per the World Health Organization (WHO), pneumonia contributes to approximately 4 million deaths worldwide annually, mostly in the high-risk cohort consisting of infants, elderly and those with myriad underlying chronic diseases (e.g., patients with surgical or functional asplenia, organ transplant patients, chronic diseases, and those with primary or secondary immunodeficiencies e.g. Acquired Immunodeficiency Syndrome [AIDS]) [9–13]. Globally, a large proportion of pneumonia cases in the adult population can be attributed to community-acquired and hospital-acquired variants. Irrespective of its type and origin, pneumonia leads to a high healthcare resource utilization (HCRU) and out-of-pocket expenditures on health [14–16]. As per the Global Burden of Disease study 2016, an estimated death rate of 187.8 per 100,000 individuals has been associated with lower respiratory infections in the Middle East region [17]. In United Arab Emirates (UAE), lower respiratory infections have been ranked among the top 10 leading causes of premature deaths in 2017 [18]. Reports suggest that pneumonia is a growing public health concern in the adult population in the Gulf countries [19, 20]. In the UAE, an annual hospital admission rate of 760 per million population has been reported for pneumonia, with a mortality rate of 13-24% [19].

Pneumococcal vaccination is an important preventive measure that has been widely used to protect against pneumococcal pneumonia (caused by *Streptococcus pneumoniae*). There are two types of pneumococcal vaccines—pneumococcal conjugate vaccine and pneumococcal polysaccharide vaccine, that are licensed for worldwide use [21–23]. Besides administration in children as part of their routine immunization, these vaccines are also highly recommended for adults at a high-risk of developing pneumococcal infections or its complications. Although, the effectiveness of these vaccines in reducing the burden of pneumonia and other life-threatening invasive pneumococcal diseases, like meningitis, bacteremia and sepsis has been substantially proven [24, 25], the use of pneumococcal vaccines in the adult population has remained sub-optimal in many countries [21, 22], including countries in the Middle East [19, 20]. The vaccination coverage in the adult population in the UAE is sub-optimal, despite recommendation by the Dubai Health Authority (DHA) [20]. This is also consistent with the United States (US), wherein only 23% of the pneumococcal vaccination coverage was noted among adults (19–64 years of age) at an increased risk of pneumonia [26], which is substantially below the US Healthy People 2020 objective of at least 60% coverage for pneumonia in these risk groups [26]. Particularly, in the Gulf countries, the factors that could majorly impact the vaccine uptake are the policies of the insurance companies towards reimbursement of the costs incurred, and a lack of awareness among the masses about the pneumococcal disease burden and the potential benefits of vaccination [20]. To establish the merit of promoting the use of pneumococcal vaccine among the adult population, further data on pneumonia burden among adults (of different risk groups) and the ensuing economic impact are warranted. Therefore, an urgent need was felt to address this knowledge gap pertaining to the adult pneumonia patients and their HCRU.

The objectives of the current study were to assess the pneumonia burden, related HCRU and costs in the adult patients with chronic diseases or immunocompromised conditions in Dubai, UAE, by using retrospective data from the Dubai Real-World Claims Database (DRWD). The findings from this study will inform management of pneumonia in the region and provide guidance to the insurance providers to update their policies and maximize treatment benefits.

## Methods

### Study design

**Fig 1:**
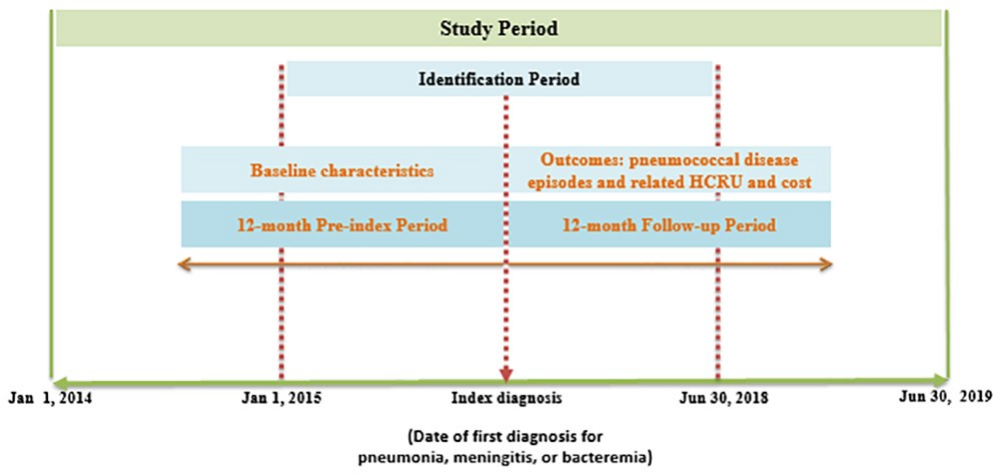
Study periods and timelines. Index diagnosis is defined as the date of first diagnosis for pneumonia. HCRU: healthcare resource utilization.

This study was a retrospective cohort analysis of patients in the DRWD claims database. The DRWD claims database is an anonymous patient-level database of all insurance claims generated from the private healthcare sector in the Emirates of Dubai. The database contains information on patient demographics, diagnoses, procedures (medical, surgical, and diagnostic), prescriptions, and other related services. Data for this study were extracted for the period from January 1, 2014 to June 30, 2019. The index diagnosis date (IDD) for each patient was defined as the date on which the first diagnosis (inpatient/outpatient/emergency) for pneumonia was made during the study identification period (between January 1, 2015 to June 30, 2018). With respect to the IDD, the following two periods were defined for each patient: 1) pre-index or baseline period (12 months prior to the IDD), and 2) follow-up period (12 months following the IDD). The study periods are depicted graphically in Fig 1. Baseline data of the eligible patients were extracted from the records collected during the pre-index period. The outcome data following the first claim for pneumonia were collected during the 12-month follow-up period.

The following inclusion criteria were used for patient selection: 1) adults aged ≥18 years at the time of index diagnosis; 2) having at least one medical claim for pneumonia (as per International Classification of Diseases, 10^th^ Revision, Clinical Modification [ICD-10-CM] codes) during the identification period. Patients with any claims for pneumonia during the pre-index period were excluded from the analysis. The records of patients meeting study eligibility criteria were categorized into three cohorts based on the health risks associated with their underlying diseases (low-risk, medium-risk, and high-risk) [15, 27, 28], determined during the pre-index period. The high-risk cohort included any patient having a diagnosis for immunocompromised conditions, such as chronic renal disease, cancer, functional or anatomic asplenia, HIV-AIDS, and organ transplantation. The medium-risk cohort included any patient with a diagnosis of chronic heart disease, diabetes mellitus and chronic liver disease, but without any diagnosis for high-risk conditions. The remaining eligible patients were included in the low-risk cohort. The study was conducted in accordance with all the local, legal and regulatory guidelines. No patient identity or medical records were disclosed for the purpose of this study, except in compliance with the applicable laws.

### Baseline covariates

Data regarding the demographics and baseline clinical characteristics were captured from the pre-index period claim records. Additionally, pre-existing comorbidities were assessed from the pre-index period records, by identifying clinically relevant disease conditions using the ICD-10 CM codes. The comorbidities were quantified using the Deyo-Charlson Comorbidity Index (CCI) score [29, 30]. Data for the baseline all-cause HCRU and healthcare costs were also captured from the pre-index period records. The all-cause HCRU and healthcare costs was stratified based type of visits (inpatient, outpatient and emergency).

### Outcome variables

There were two outcomes of interest for this study: 1) the number of pneumonia episodes, and 2) HCRU and the associated healthcare costs. The healthcare costs were calculated using the dollar value of 2020. The data regarding these outcomes were extracted from the follow-up period claims records. The number of pneumonia episodes equaled the number of pneumonia claims during follow-up period. The first claim pertaining to pneumonia submitted during the identification period was considered the index pneumonia episode. The date of the first such claim was considered the start date of the first pneumonia episode and the subsequent claims were considered under the same pneumonia episode unless there was an interval of 30 days between the two consecutive claims. The pneumonia episode data were stratified on the basis of age into 18–29, 30–39, 40–49, 50–59, and ≥60 years subgroups. The HCRU and associated costs were calculated based on the number of claims related to office visits, emergency room (ER) visits, hospitalizations and pharmacy claims during the follow-up period. The all-cause and pneumonia-related HCRU and costs were determined. The all-cause data were extracted from all claims (for all diseases/conditions [including pneumonia]) available during the follow-up period. The pneumonia-related data were collected specifically from pneumonia claims available during the follow up. These data (all-cause and pneumonia) were stratified based on the type of claims i.e, inpatient, outpatient, and emergency claims, and the type of medical support availed (drugs, medical services and medical procedures coded using Current Procedural Terminology [CPT] and Healthcare Common Procedure Coding System [HCPCS] codes) during the 12-month follow-up period.

### Statistical analysis

Descriptive statistics were calculated for all the study variables assessed during the pre-index and follow-up periods: demographics and clinical characteristics, comorbidities, risk factors, number of pneumonia episodes, healthcare utilization and costs (all-cause and pneumonia specific). The number and percentages were provided for dichotomous and polychotomous variables. The continuous variables were summarized as mean and standard deviation wherever applicable. The stratification of patients based on underlying comorbidities into low, medium and high-risk groups, allowed to exclude the confounding factors (unrelated to pneumonia).

## Results

### Demographic and clinical characteristics

**Fig 2:**
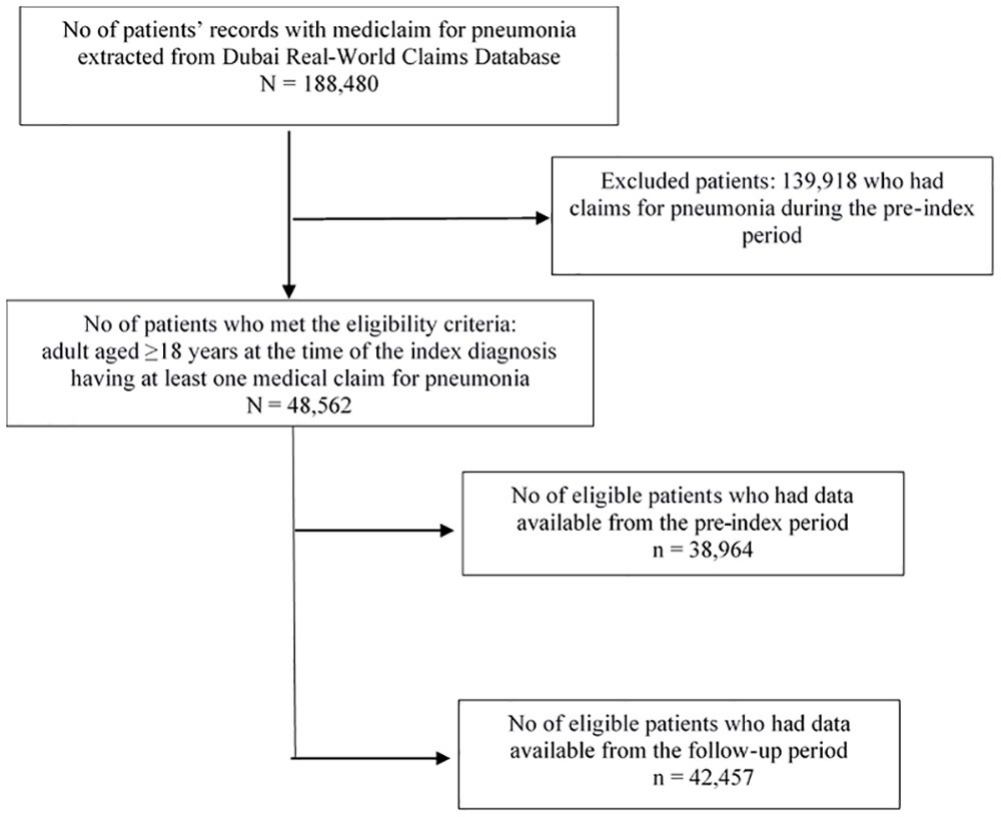
Patient disposition flow chart. Index diagnosis is defined as the date of first diagnosis for pneumonia; pre-index period is the 12-month period prior to index diagnosis; follow-up period is the 12-month period after index diagnosis.

The records of 188,480 patients were extracted from the database, of which, records of 48,562 patients met the eligibility criteria and were included in the analyses. Among the eligible patients, 38,964 (80.2%) patients had data available from the pre-index period, while 42,457 (87.4%) patients had data from the follow-up period. Patient disposition is presented in Fig 2.

The demographics and clinical characteristics of the selected patient population at baseline are presented in Table 1. Most of the eligible patients were men (65.6%). The mean age was 39.9 years, with six out of every seven (85.9%) patients belonging to the age group of 18–50 years, both inclusive. Of the eligible patients, 62.1% (n = 30,158), 36.2% (n = 17,567) and 1.7% (n = 837) belonged to the low-risk, medium-risk, and high-risk cohorts, respectively. Overall, the most frequently noted comorbidities at baseline were related to chronic pulmonary disease (24.7%). The mean number of HCRU claims made during the pre-index period was 10.9 per patient and the associated mean healthcare cost was 1,327 United States Dollar (USD) per patient. Overall, the highest number of claims were made for the outpatient visits (mean: 10.6 claims), however, the largest proportion of the incurred costs was attributed to the claims for the inpatient visits (mean: 4,377 USD); similar observations were made for all the risk cohorts. Among the risk cohorts, the highest number of claims and the highest costs recorded for overall and each type of visits during the pre-index period were noted for the high-risk cohort (overall mean claims was 22.8 and overall mean cost was 8,881 USD) (Table 1).

**Table 1:** Baseline demographics, clinical characteristics, healthcare resource utilization and associated costs during pre-index period (patients having at least one claim for pneumonia during identification period were included in the analysis; Dubai Real-World Claims Database, 2014 to 2019).

	Overall cohort (n = 48,562)		High-risk cohort (n = 837)		Medium-risk cohort (n = 17,567)		Low-risk cohort (n = 30,158)	
	n	%	n	%	n	%	n	%
Age, years								
**18–50**	41,738	85.9%	497	59.4%	13,815	78.6%	27,426	90.9%
**51–60**	4,787	9.9%	171	20.4%	2,546	14.5%	2,070	6.9%
**>60**	2,037	4.2%	169	20.2%	1,206	6.9%	662	2.2%
Mean age (SD)	39.9 (10.1)		48.8 (13.7)		42.6 (10.7)		38.1 (9.1)	
Gender								
**Men**	31,879	65.6%	548	65.5%	11,815	67.3%	19,516	64.7%
Deyo-Charlson Comorbidity Index (CCI) scores								
**0**	29,185	60.1%	2	0.2%	0	0	29,183	96.8%
**1–2**	16,443	33.9%	224	26.8%	15,258	86.9%	961	3.2%
**3–4**	2,364	4.9%	335	40.0%	2,015	11.5%	14	0
**5–6**	430	0.9%	163	19.5%	267	1.5%	0	0
**7+**	140	0.3%	113	13.5%	27	0.2%	0	0
Comorbidities								
**Chronic pulmonary disease**	11,991	24.7%	0	0	11,991	68.3%	0	0
**Asthma**	10,391	21.4%	0	0	10,391	59.2%	0	0
**Diabetes without complications**	6,123	12.6%	0	0	6,123	34.9%	0	0
**Mild liver disease**	3,263	6.7%	0	0	3,263	18.6%	0	0
**Diabetes with complications**	1,664	3.4%	0	0	1,664	9.5%	0	0
**Renal disease**	482	1.0%	482	57.6%	0	0	0	0
**Cancer**	361	0.7%	361	43.1%	0	0	0	0
**Other comorbidites** *	665	1.4%	46	5.5%	619	3.5%	0	0
All-cause Healthcare Resource Utilization: number of claims per patient per year, mean (SD)								
**Overall** ^a^	10.9 (11.7)		22.8 (25.4)		13.7 (13.2)		8.5 (8.7)	
**Inpatient**^b^	1.5 (1.4)		2.4 (4.3)		1.4 (0.8)		1.4 (0.7)	
**Outpatient**^c^	10.6 (11.3)		21.7 (23.1)		13.3 (12.8)		8.2 (8.4)	
**Emergency**^d^	2.6 (3.3)		3.7 (9.6)		2.8 (3.1)		2.4 (2.4)	
All-cause Healthcare Cost per patient per year, USD, mean (SD)								
**Overall** ^a^	1,327 (4,326)		8,881 (23,078)		1,584 (3,001)		878 (2,346)	
**Inpatient**^b^	4,377 (10,008)		13,312 (27,385)		4,030 (6,650)		3,363 (6,463)	
**Outpatient**^c^	1,019 (2,859)		5,744 (16,213)		1,275 (1,925)		669 (1,271)	
**Emergency**^d^	316 (443)		527 (928)		337 (438)		283 (382)	

^a^ n = 38,964 for overall cohort; n = 777 for high-risk cohort; n = 15,982 for medium-risk cohort; n = 22,205 for low-risk cohort.

^b^n = 2,500 for overall cohort; n = 181 for high-risk cohort; n = 1,098 for medium-risk cohort; n = 1,221 for low-risk cohort.

^c^n = 38,712 for overall cohort; n = 766 for high-risk cohort; n = 15,910 for medium-risk cohort; n = 22,036 for low-risk cohort.

^d^n = 4,275 for overall cohort; n = 172 for high-risk cohort; n = 1,803 for medium-risk cohort; n = 2,300 for low-risk cohort.

*Other comorbidities reported in <300 patients: congestive heart failure, myocardial infarction, peripheral vascular disease, metastatic carcinoma, moderate or severe liver disease, functional or anatomic asplenia, and HIV/AIDS.

Number of comorbidities categorized as “others”: overall, 30,158 (62.1%); low-risk cohort, 30,158 (100%); high and medium-risk cohorts, 0.

SD: standard deviation; USD: United States Dollar. All healthcare costs were calculated using the dollar value of 2020.

### Pneumonia episodes

Of the total eligible population (n = 48,562), 29.4% (n = 14,254) patients had at least one pneumonia episode during the 12-month follow-up period. Of these, 75.8% (n = 10,810) patients had a solitary pneumonia episode. The mean annual pneumonia incidence rate was 1.3 episodes, with a largely similar distribution noted across all the risk cohorts (1.2, high risk, 1.3, medium risk, 1.3, low risk). There was no particular trend observed in the incidence of pneumonia episodes based on the patient’s risk status (high/medium/low). The percentage of patients with 1 pneumonia episode was similar across all risk groups (79.3%, high risk, 75.2% medium risk, 76.2% low risk) (Table 2). Stratification by age revealed that most of the patients with pneumonia episodes belonged to the age group of 30–49 years (approximately 70%), both inclusive (Table 2).

**Table 2:** Pneumonia episodes in age-stratified populations (patients having at least one claim for pneumonia during 12-month follow-up period were included in the analysis; Dubai Real-World Claims Database, 2014 to 2019).

Pneumonia Episode Counts						
		1	2	3	4	>5
		Number of patients with pneumonia episodes, n (%)				
Age groups, (years)	Cohorts					
**All patients**	Overall	10,810 (75.84)	3,062 (21.48)	323 (2.27)	47 (0.33)	12 (0.08)
	High-risk	307 (79.33)	72 (18.60)	7 (1.81)	1 (0.26)	0 (0.00)
	Medium-risk	4,526 (75.17)	1,305 (21.67)	158 (2.62)	22 (0.37)	10 (0.17)
	Low-risk	5,977 (76.18)	1,685 (21.48)	158 (2.01)	24 (0.31)	2 (0.03)
**18–29**	Overall	1,084 (10.03)	346 (11.30)	33 (10.22)	3 (6.38)	1 (8.33)
	High-risk	10 (3.26)	2 (2.78)	1 (14.29)	0 (0.00)	0 (0.00)
	Medium-risk	314 (6.94)	96 (7.36)	13 (8.23)	1 (4.55)	1 (10.00)
	Low-risk	760 (12.72)	248 (14.72)	19 (12.03)	2 (8.33)	0 (0.00)
**30–39**	Overall	4,299 (39.77)	1,264 (41.28)	145 (44.89)	22 (46.81)	2 (16.67)
	High-risk	48 (15.64)	16 (22.22)	1 (14.29)	0 (0.00)	0 (0.00)
	Medium-risk	1,523 (33.65)	473 (36.25)	63 (39.87)	8 (36.36)	1 (10.00)
	Low-risk	2,728 (45.64)	775 (45.99)	81 (51.27)	14 (58.33)	1 (50.00)
**40–49**	Overall	3,218 (29.77)	904 (29.52)	87 (26.93)	13 (27.66)	3 (25.00)
	High-risk	80 (26.06)	17 (23.61)	2 (28.57)	0 (0.00)	0 (0.00)
	Medium-risk	1,440 (31.82)	422 (32.34)	49 (31.01)	5 (22.73)	2 (20.00)
	Low-risk	1,698 (28.41)	465 (27.60)	36 (22.78)	8 (33.33)	1 (50.00)
**50–59**	Overall	1,369 (12.66)	361 (11.79)	40 (12.38)	7 (14.89)	2 (16.70)
	High-risk	64 (20.85)	22 (30.56)	2 (28.57)	1 (100.00)	0 (0.00)
	Medium-risk	747 (16.50)	208 (15.94)	23 (14.56)	6 (27.27)	2 (20.00)
	Low-risk	558 (9.34)	131 (7.77)	15 (9.49)	0 (0.00)	0 (0.00)
**≥60**	Overall	840 (7.77)	187 (6.11)	18 (5.57)	2 (4.26)	4 (33.33)
	High-risk	105 (34.20)	15 (20.83)	1 (14.29)	0 (0.00)	0 (0.00)
	Medium-risk	502 (11.09)	106 (8.12)	10 (6.33)	2 (9.09)	4 (40.00)
	Low-risk	233 (3.90)	66 (3.92)	7 (4.43)	0 (0.00)	0 (0.00)

Total number of patients with at least 1 pneumonia episode in 12-month follow-up: overall cohort, n = 14,254; high-risk cohort, n = 387; medium-risk cohort, n = 6,021; low-risk cohort, n = 7,846.

### Healthcare resource utilization and associated costs

The HCRU and the associated gross costs after the IDD for the all-cause and the pneumonia-related claims assessed for 42,457 and 14,254 patients, respectively, who had data available from the follow-up period, are presented here:

Overall, the mean number of all-cause HCRU claims and the associated mean costs per patient were 11.6 and 1,947 USD, respectively (Table 3). Overall, about >45% increase in all-cause healthcare costs was observed in the post-pneumonia period, as compared to the costs incurred during the year before the first pneumonia episode (mean cost: 1,327 USD). Among the risk cohorts, both the highest mean number of claims and the associated costs were noted for patients in the high-risk cohort (mean HCRU claims = 22.0 per patient; mean cost = 14,184 USD). On the other hand, the mean number of pneumonia related HCRU claims and the associated costs per patient were 2.0 and 1,305 USD, respectively (Table 4). The mean number of pneumonia-related HCRU claims did not vary across the risk cohorts (mean number of claims: high-risk = 1.9 per patient, medium-risk = 2.2 per patient and low-risk = 1.9 per patient), but the associated mean costs per patient for the high-risk cohort (10,207 USD) was about 8–11 times higher compared to the mean costs per patient for the medium-risk (1,283 USD) and the low-risk (882 USD) cohorts.

**Table 3:** Healthcare resource utilization and healthcare cost per patient (all-cause claims) during 12-month follow-up (patients having at least one claim for pneumonia during 12-month follow-up period were included in the analysis; Dubai Real-World Claims Database, 2014 to 2019).

		Overall Cohort	High-risk Cohort	Medium-risk Cohort	Low-risk Cohort
**Overall population**		**n = 42,547**	**n = 782**	**n = 16,113**	**n = 25,562**
	**Healthcare resource utilization rate per patient, mean (SD)**	11.6 (12.4)	22.0 (29.7)	13.9 (13.6)	9.9 (10.1)
	**Gross cost per patient (USD), mean (SD)**	1,947 (7,899)	14,184 (40,331)	2,240 (70,022)	1,388 (4,234)
**Inpatient**		**n = 6,076**	**n = 305**	**n = 2,560**	**n = 3,211**
	**Healthcare resource utilization rate per patient, mean (SD)**	1.3 (1.3)	2.2 (5.0)	1.3 (0.8)	1.3 (0.7)
	**Gross cost per patient (USD), mean (SD)**	4,901 (17,456)	20,195 (55,210)	4,609 (15,095)	3,681 (9,088)
**Outpatient**		**n = 41,635**	**n = 710**	**n = 15,833**	**n = 25,092**
	**Healthcare resource utilization rate per patient, mean (SD))**	11.4 (12.0)	22.8 (28.4)	13.7 (13.3)	9.6 (9.8)
	**Gross cost per patient (USD), mean (SD)**	1,232 (3,244)	6,831 (16,257)	1,496 (2,617)	908 (2,154)
**Emergency**		**n = 4,550**	**n = 136**	**n = 1,717**	**n = 2,697**
	**Healthcare resource utilization rate per patient, mean (SD)**	2.3 (2.5)	2.5 (2.4)	2.4 (2.7)	2.3 (2.4)
	**Gross cost per patient (USD), mean (SD))**	347 (474)	609 (755)	352 (512)	331 (425)

SD: standard deviation; USD: Unites States Dollar. All healthcare costs were calculated using the dollar value of 2020.

**Table 4:** Healthcare resource utilization and healthcare cost per patient (pneumonia-related claims) during 12-month follow-up (patients having at least one claim for pneumonia during 12-month follow-up period were included in the analysis; Dubai Real-World Claims Database, 2014 to 2019).

		Overall Cohort	High-risk Cohort	Medium-risk Cohort	Low-risk Cohort
**Overall population**		**n = 14,254**	**n = 387**	**n = 6,021**	**n = 7,846**
	**Healthcare resource utilization rate per patient, mean (SD)**	2.0 (1.8)	1.9 (2.5)	2.2 (2.0)	1.9 (1.5)
	**Gross cost per patient (USD), mean (SD)**	1,305 (9,132)	10,207 (34,963)	1,283 (9,127)	882 (4,832)
**Inpatient**		**n = 3,430**	**n = 221**	**n = 1,476**	**n = 1,733**
	**Healthcare resource utilization rate per patient, mean (SD)**	1.1 (0.3)	1.2 (0.6)	1.1 (0.4)	1.1 (0.3)
	**Gross cost per patient (USD), mean (SD)**	4,818 (18,141)	17,458 (44,913)	4,560 (18,014)	3,426 (9,829)
**Outpatient**		**n = 11,830**	**n = 210**	**n = 5,010**	**n = 6,610**
	**Healthcare resource utilization rate per patient, mean (SD)**	2.0 (1.7)	2.2 (3.1)	2.2 (1.9)	1.9 (1.5)
	**Gross cost per patient (USD), mean (SD)**	166 (374)	425 (1935)	190 (318)	140 (229)
**Emergency**		**n = 491**	**n = 14**	**n = 204**	**n = 273**
	**Healthcare resource utilization rate per patient, mean (SD)**	1.7 (1.4)	1.9 (1.1)	1.7 (1.6)	1.6 (1.1)
	**Gross cost per patient (USD), mean (SD)**	206 (236)	161 (137)	201 (233)	212 (242)

SD: standard deviation; USD: United States Dollar. All healthcare costs were calculated using the dollar value of 2020.

The mean all-cause and pneumonia-related costs were largely attributed to the claims for inpatient visits in overall and in each of the risk cohorts (Tables 3 and 4). A large proportion of all-cause HCRU costs was attributed to claims pertaining to CPT and HCPCS codes across the risk cohorts (up to approximately 5,059 USD) (Table 5). In the high-risk cohort, claims for drugs and services also contributed largely to the gross expenditures. The pneumonia-related HCRU costs were mostly incurred from the claims related to CPT codes and medical services availed in overall and across all the risk cohorts (Table 5).

**Table 5:** Healthcare cost per patient based on activity type (all-cause and pneumonia-related) during 12-month follow-up (patients having at least one claim for pneumonia during 12-month follow-up period were included in the analysis; Dubai Real-World Claims Database, 2014 to 2019).

	All-cause healthcare cost per patient (net cost)				Pneumonia-related healthcare cost per patient (net cost)			
	Overall Cohort n = 42,457	High-risk Cohort n = 782	Medium-risk Cohort n = 16,113	Low-risk Cohort n = 25,562	Overall Cohort n = 14,254	High-risk Cohort n = 387	Medium-risk Cohort n = 6,021	Low-risk Cohort n = 7,846
**Activity types**								
**Drugs**								
**n**	38,798	742	15,014	23,042	10,613	292	4,514	5,807
**Mean (SD), USD**	482 (2,265)	3,948 (11,495)	627 (1,754)	277 (1,394)	244 (1,378)	1,815 (5,811)	244 (1,096)	165 (846)
**CPT codes**								
**n**	36,675	737	14,533	21,405	10,065	318	4,409	5,338
**Mean (SD), USD**	875 (2,721)	5,059 (12,847)	935 (2,278)	690 (1,687)	551 (2,709)	3,350 (9,231)	542 (2,642)	392 (1,586)
**HCPCS codes**								
**n**	4,207	227	1,784	2,196	1,243	100	564	579
**Mean (SD), USD**	566 (2,147)	2,635 (7,278)	501 (1,462)	404 (1,080)	444 (2,232)	2,235 (6,554)	376 (1,519)	200 (694)
**Services**								
**n**	37,889	731	14,696	22,462	6,622	222	2,793	3,607
**Mean (SD), USD**	423 (1,835)	2,467 (9,859)	443 (1,440)	344 (1,010)	581 (2,657)	3,624 (9,756)	551 (2,390)	416 (1,454)
**Unknown**								
**n**	8,662	351	3,869	4,442	4,101	223	1,788	2,090
**Mean (SD), USD**	4945 (2,331)	2,158 (8,633)	430 (1,093)	421 (1,859)	743 (2,606)	2,523 (9,675)	665 (1,274)	620 (1,280)

CPT: Current Procedural Terminology; HCPCS: Healthcare Common Procedure Coding System; SD: standard deviation; USD: United States Dollar. All healthcare costs were calculated using the dollar value of 2020.

## Discussion

The rising burden of pneumonia in the Gulf countries is a point of concern [19, 20, 31, 32], and there is a paucity of epidemiological reports that reflect upon the clinical and economic burden of pneumonia on the healthcare sector in these countries. To our knowledge, the current study is the first attempt to compare the pre- and post-pneumonia HCRU and healthcare costs among the adults in Dubai, UAE. In this study, an overall >45% increase in the all-cause healthcare costs was observed in the post-pneumonia period (mean cost: 1,947 USD), as compared to the costs incurred during the year prior to the first pneumonia episode (mean cost: 1,327 USD). This finding highlights the financial implications associated with this malady. However, the overall pattern for HCRU remained largely similar during the pre- and post-pneumonia periods.

Intriguingly, of all the claims that were recorded in the identification period, approximately 86% were from the relatively younger population (18–50 years). Also, approximately 80% of the medium-risk cohort was constituted by the younger population. This is aligned with the high prevalence of smoking and respiratory illnesses, the known potential risk factors for pneumonia infections, among the younger population (age below 40 years) in the Middle East countries [33]. Furthermore, the incidences of viral infections are high in the UAE, with approximately 50% of the patients visiting a general physician, suffering from a viral infection [34]. These patients are at a high-risk of developing severe bronchial pneumonia resulting in hospitalization. A report showed that influenza virus (flu virus) was the most common respiratory virus in all the age groups in the UAE [35]. There are studies indicating that mixed viral-bacterial infections, like combinations of *Streptococcus pneumoniae* with various respiratory viruses like rhinovirus or influenza, have been associated with severe pneumonia and mortality in adults [8].

Surprisingly, the overall pneumonia incidence rates were largely similar across the risk cohorts. Similarly, the mean number of pneumonia-related HCRU claims were also consistent across the risk cohorts. Despite having the similar number of claims, the mean pneumonia-related expenditures were found to be much higher in the high-risk cohort than the other cohorts. The overall costs for the high-risk cohort were almost 8–11 times higher than the costs for the low/medium-risk cohorts. This may be because the patients categorized into the high-risk cohort had severe underlying comorbidities, therefore requiring extensive inpatient medical care compared to the low/medium-risk cohorts, leading to an overall increase in the expenditures. These findings are consistent with various prior publications that highlight the high economic burden of pneumonia management in adults with severe underlying comorbidities [12, 15, 36]. Furthermore, the study data revealed a huge difference in the costs incurred from the inpatient and the outpatient visits in all the risk cohorts, with approximately 20–30 times additional costs associated with the inpatient visits. Notably, the costs related to the emergency visits were very low in the pneumonia population in Dubai. Overall, these findings were similar to the previously published reports that have shown that the inpatient care accounts for about 90% of the overall pneumonia-related expenditures [12, 37].

Thus, the findings of this study re-emphasize the high economic burden of pneumonia in the high-risk adult population. Furthermore, the high occurrences of pneumonia infection among the younger population, which comprises a large proportion of the working class and socially active group, may increase the chances of the infection spreading at a much faster rate, putting the vulnerable section of the society, such as the elderly and immunocompromised individuals, at a higher risk of contracting infection and thus subjecting them to high financial implications.

These findings, therefore, allude towards the importance of pneumococcal immunization, along with other preventive measures, for adults, especially those at a high-risk of contracting pneumococcal infection. The DHA recommends vaccination for the adults at a high-risk of pneumococcal infections, in line with the WHO guidelines which recommends that all the immunocompromised adults should receive pneumococcal vaccine. As per the Center of Disease Control and Prevention (CDC) report, a significant decline in the invasive pneumococcal disease cases (50 to 60%) has been noticed in the US, following the introduction of pneumococcal vaccines [36]. Despite the health authority recommendations, pneumococcal immunization coverage among the adults in the Gulf region, including Dubai, remains low (as per empirical reports). Hence, efforts are required to increase the awareness about pneumococcal immunization among the adults in this region. Additionally, insurance coverage for the adult pneumococcal vaccination may go a long way in increasing the uptake of the vaccine and subsequently saving substantial HCRU costs associated with pneumonia among adults.

The strength of our study is that we used data from DRWD which is the largest source of secondary data on pneumonia in Dubai. It captures information for almost the entire population covered by the Dubai private health insurance, predominantly comprising the multi-ethnic expatriate population. Therefore, the results of this study would have a good level of generalizability and reflect on the real-life patterns in the pneumonia prevalence and its related burden on the healthcare system and the financial infrastructure in Dubai. Further, since we used the claims data, there is a less possibility of selection bias and the data are inclusive of all the treatment modalities performed by all the healthcare providers and medical specialties [38]. These data would help in increasing the transparency and effectiveness of the healthcare system in better handling the growing issues of infectious diseases in the region.

There are a few limitations that need to be acknowledged. In this study, the immediate first pneumonia claim after the IDD was considered as the first pneumonia episode during the follow-up period and the claims thereafter were considered as distinct pneumonia episodes, if the interval period between any consecutive claims was 30 days. The interval period of 30 days used in this study was much shorter compared to the interval period between the two consecutive claims used in other published studies, ranging from 60–90 days [15, 39]. This may be the potential reason for similar pneumonia incidence rates observed across the low, medium, and high-risk cohorts in the study. Further, considering this was a retrospective analysis of claims data, there are few limitations that might influence interpretation of the study data. For example, information regarding the diagnoses codes in Mediclaim may not always be accurate and might result in misclassification of outcomes, which can further jeopardize the internal validity of the study. Nevertheless, we believe that the impact of this limitation in the current study would be similar to the other global studies. Furthermore, claims database studies are generally limited by the lack/insufficiencies of important clinical data. Despite these limitations, the real-world study provides valuable insights about the pneumonia patients in Dubai and would aid clinical decision-making in the pneumonia management in the UAE.

## Conclusions

The analyses of the claims data showed that the overall burden of pneumonia was high in Dubai, UAE. The healthcare costs were especially high in individuals with severe underlying comorbidities. Overall, our findings would help clinicians and insurance policy makers to make informed decisions to improvise future treatment approaches for pneumonia in Dubai, UAE.

## Supporting information

S1 Checklist STROBE. (DOCX)

S1 Data Data analysis. (XLSX)

## Abbreviations

CCI: Deyo-Charlson Comorbidity Index
CPT: Current Procedural Terminology
DHA: Dubai Health Authority
DRWD: Dubai Real-World Claims Database
ER: Emergency Room
HCPCS: Healthcare Common Procedure Coding System
HCRU: Healthcare Resource Utilization
IDD: Index Diagnosis Date
UAE: United Arab Emirates
USD: United States Dollar
WHO: World Health Organization
